# ROBUST BIOMARKERS OF AGING AND THEIR BIOLOGICAL ORIGIN

**DOI:** 10.1093/geroni/igz038.131

**Published:** 2019-11-08

**Authors:** Dmitriy Podolskiy, Dmitriy Podolskiy, Vadim Gladyshev

**Affiliations:** Brigham & Women’s Hospital, Boston, Massachusetts, United States

## Abstract

The biological origin of impressive accuracy of DNA methylation clocks, the most precise currently available biological markers of aging in humans and model animals, remains largely unclear. In addition, they sometimes suffer from uncontrollable precision loss out of sample. To address these two issues, we develop a novel method for constructing robust molecular markers of age based on network analysis of age-dependent omics data. The newly developed robust markers of aging in yeast, fruit fly, mouse and human are nearly free of batch effects and have a transparent biological nature related to tight control of translation-related processes and their disregulation associated with aging.

